# Hfq affects mRNA levels independently of degradation

**DOI:** 10.1186/1471-2199-11-17

**Published:** 2010-02-18

**Authors:** Jacques Le Derout, Irina V Boni, Philippe Régnier, Eliane Hajnsdorf

**Affiliations:** 1 UPR CNRS n° 9073, conventionnée avec l'Université Paris 7 - Denis Diderot Institut de Biologie Physico-Chimique, 13 rue Pierre et Marie Curie, 75005 Paris, France; 2 Shemyakin-Ovchinnikov Institute of Bioorganic Chemistry, Russian Academy of Sciences, 117997 Moscow, Russia

## Abstract

**Background:**

The bacterial Lsm protein, Hfq, is an RNA chaperone involved in many reactions related to RNA metabolism, such as replication and stability, control of small RNA activity and polyadenylation. Despite this wide spectrum of known functions, the global role of Hfq is almost certainly undervalued; its capacity to bind DNA and to interact with many other proteins are only now beginning to be taken into account.

**Results:**

The role of Hfq in the maturation and degradation of the *rpsO *mRNA of *E. coli *was investigated *in vivo*. The data revealed a decrease in *rpsO *mRNA abundance concomitant to an increase in its stability when Hfq is absent. This indicates that the change in mRNA levels in *hfq *mutants does not result from its modification of RNA stability. Moreover, a series of independent experiments have revealed that the decrease in mRNA level is not a consequence of a reduction of translation efficiency and that Hfq is not directly implicated in translational control of *rpsO *expression. Reduced steady-state mRNA levels in the absence of Hfq were also shown for *rpsT, rpsB *and *rpsB-tsf*, but not for *lpp, pnp *or tRNA transcripts. The abundance of chimeric transcripts *rpsO-lacZ *and *rpsB-lacZ*, whose expression was driven by *rpsO *and *rpsB *promoters, respectively, was also lower in the *hfq *null-mutants, while the β-galactosidase yield remained about the same as in the parent wild-type strain.

**Conclusions:**

The data obtained suggest that alteration of *rpsO, rpsT *and *rpsB-tsf *transcript levels observed under conditions of Hfq deficiency is not caused by the post-transcriptional events, such as mRNA destabilization or changes in translation control, and may rather result from changes in transcriptional activity. So far, how Hfq affects transcription remains unclear. We propose that one of the likely mechanisms of Hfq-mediated modulation of transcription might operate early in the elongation step, when interaction of Hfq with a nascent transcript would help to overcome transcription pauses and to prevent preliminary transcript release.

## Background

Hfq is an RNA binding protein initially identified as a host factor required for the replication of the phage Qβ RNA [1]. It was then demonstrated to belong to the Sm-like protein family involved in many RNA processing events in eukaryotes [2]. The Hfq-encoding gene is widely conserved in bacteria and found in many sequenced bacterial genomes [3]. Hfq is a highly abundant protein considered to act as a global regulator of gene expression [4,5]. It has recently received much attention because of its crucial role in diverse cellular processes controlled by small non-coding RNAs (ncRNAs), where Hfq facilitates pairing of ncRNAs with their target mRNAs [6-9]. This explains, at least partly, why inactivation of the *hfq *gene causes pleiotropic phenotypes [5]. Some of changes in the gene expression pattern are related to reduced translation efficiency of the *rpoS *mRNA, encoding the major stress sigma factor σ^S ^[4,10], others to the induction of the σ^E^-mediated envelope stress response [11-13] and the deficiency of the σ^H^-mediated cytoplasmic stress response [12].

In addition, Hfq may affect some processes through its direct interaction with RNAs, e.g. with Qβ phage RNA during replication [14,15] or with its own mRNA where it acts as a translational autorepressor [16,17]. Hfq interactions with tRNAs [18,19] and tRNA precursors [20] have also been reported.

Hfq function in the cell may also be mediated by protein-protein contacts. Hfq has been reported to interact with numerous proteins including ribosomal proteins, RNases, helicases, Rho-factor, RNA polymerase (in the presence of S1 protein), protein H-NS and poly(A)polymerase (PAP I) [21-25]. Related to the last observation, we have also shown that Hfq stimulates PAP I mediated synthesis of poly(A) tails by promoting the processivity of the enzyme and by protecting the poly(A) tails from exoribonucleolytic degradation [26-29]. Finally, Hfq has also been identified as a DNA binding protein that preferentially binds curved DNA and affects negative supercoiling [5,30,31]. However, in a cell, the majority of Hfq is located in the cytoplasm, presumably in association with the translation machinery, and only a minor fraction is associated with the nucleoid [32].

In the present work, our initial goal was to investigate the role of Hfq in the maturation and degradation of the *rpsO *mRNA of *E. coli in vivo*. The degradation pathway of the *rpsO *transcript, coding for ribosomal protein S15, is one of the best understood decay-pathways in *E. coli*. The genes encoding ribosomal protein S15 (*rpsO*) and polynucleotide phosphorylase (*pnp*) occupy adjacent positions and are oriented in the same direction on the *E. coli *chromosome. The two genes have their own promoters, P1 and P2 respectively, and can be expressed as monocistronic transcripts or as an *rpsO-pnp *dicistronic transcript. In this latter case, an endonucleolytic cleavage by RNase III produces a P1-RIII *rpsO *mRNA slightly longer than the P1-t1 *rpsO *monocistronic transcript [33,34]. The initial step in the degradation of the *rpsO *mRNA is an RNase E cleavage that generates RNA molecules lacking the Rho-independent terminator, which are then rapidly degraded by the 3' to 5' exonucleases PNPase and RNase II [35-37]. The *rpsO *mRNA is stabilized by efficient translation because terminating ribosomes occlude the site for the rate-limiting RNase E cleavage located 10 nucleotides downstream of the translation termination codon [38]. Since ribosomal protein S15 autoregulates its synthesis at the posttranscriptional level by repressing its own translation [39] and thereby decreasing the number of ribosomes translating the *rpsO *mRNA, it has been proposed that the coupling of the mRNA stability to translation allows the cell to adapt the amount of the *rpsO *mRNA to the need for ribosomal protein S15. Besides the RNase E-mediated pathway, exonucleolytic poly(A)-dependent degradation also plays a significant role, which becomes predominant when RNase E is inactivated [40-42]. Although the regulation of the *rpsO *gene expression was studied primarily at the posttranscriptional level, its transcription is most likely also modulated. However, while transcriptional start points have been identified precisely [33], transcriptional control remains largely unexplored.

Experiments were initiated to examine whether Hfq interferes with the *rpsO *mRNA decay mediated by RNase E or polyadenylation *in vivo*, as was previously shown *in vitro *[29]. Unexpectedly, we found that Hfq deficiency induces a decrease in *rpsO *mRNA abundance concomitant to an increase in its stability. We present here a set of data suggesting that in the case of *rpsO *and some other cases, modulation of gene expression observed upon Hfq deficiency may result from changes in transcriptional yield.

## Results

### Inactivation of *hfq *reduces the level of the *rpsO *mRNA

**Figure 1:**
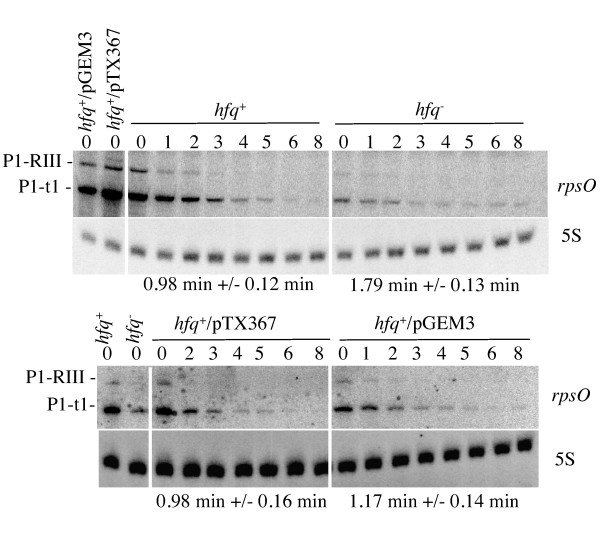
**Effects of Hfq inactivation on the stability and the abundance of the *rpsO *transcripts**. Comparison of the decay-rate of the *rpsO *mRNA in strains N3433 (wt), IBPC929 (*hfq*^-^), N3433/pTX367 and N3433/pGEM3. Cultures were grown at 37°C to OD_600_≈0.4, RNA was extracted from aliquots withdrawn after addition of rifampicin (500 μg/ml) (time intervals in min are indicated above the lanes) and then subjected to Northern blot analysis. The blots were hybridized with the *rpsO *probe and then with the probe for 5S RNA to normalize the RNA content per lane. RNA levels were quantified using a PhosphoImager; half-lives are indicated below each autoradiograph. Antibiotics were added when required.

To determine whether Hfq affects the abundance and the decay-rate of the *rpsO *mRNA *in vivo*, we determined the stability of the corresponding transcripts in a set of isogenic wt (*hfq*^+^), *hfq*^- ^and Hfq overproducing strains containing pTX367 [5], a pGEM3 derivative expressing the *hfq *gene from its own promoter. The decay-rate of the *rpsO *mRNA was measured after inhibition of transcription by rifampicin. The more abundant mRNA species detected on Northern blots are the 420 nucleotide monocistronic transcript (P1-t1) and the 502 nucleotide RNase III-processed *rpsO-pnp *mRNA (P1-RIII). Fig. 1 shows that amounts of both *rpsO *transcripts are more abundant in the *hfq*^+ ^strain than in the *hfq*^- ^mutant. There is in fact 10 times more P1-t1 mRNA in the wild type strain than in the *hfq *mutant, and 2.2 times more in *hfq*^+^/pTX367 overproducing cells than in the wild type bacteria transformed with the empty vector (compare time 0 in rifampicin experiments). Surprisingly, this drop in intracellular concentration is associated with stabilization expected to cause an accumulation of the *rpsO *transcript. Indeed the half-life of the *rpsO *mRNA is significantly longer in the *hfq *mutant (1.79 +/- 0.13 min) than in the wild type strain (0.98 +/- 0.12 min), thus confirming that Hfq can activate RNA decay [25]. The fact that no difference in stability was observed when Hfq was overproduced (half-life 0.98 +/- 0.16 min) relative to the control strain (half-life 1.17 +/- 0.14 min), suggests that the Hfq level in the wt strain is sufficient to exert a maximum destabilizing effect on the *rpsO *mRNA decay (Fig. 1 and Table 1). The data above clearly indicate that the drop in *rpsO *mRNA levels associated with Hfq deficiency does not result from its destabilization. On the contrary, we observed an increase in the *rpsO *mRNA stability, which should, in theory, result in its accumulation. It means that the drop in the *rpsO *mRNA level is, in fact, partially compensated by its stabilization.

**Table 1:** Variation and stability of the *rpsO, rpsO*Δ and *rpsT *transcripts as a function of Hfq quantity.

	*rpsO *mRNA (**)		*rpsO*Δ mRNA (**)	
Strains	t1/2 (min) (*)	Relative RNA abundance at T = 0	t1/2 (min) (*)	Relative RNA abundance at T = 0
wt	0.98+/-0.12	100		
*hfq*	1.79+/-0.13	10.3		
wt/pGEM3	1.17+/-0.14	100		
wt/pTX367	0.98+/-0.16	222		
wt/pΔS15AUG	1.13+/-0.1	100	3.16+/-0.07	100
*hfq*/pΔS15AUG	2.09+/-0.12	50	4.67+/-0.09	62.5
	*rpsT *mRNA			
Strains	t1/2 (min) (*)	Relative RNA abundance at T = 0		
wt P1-t1	1.31+/-0.17	100		
wt P2-t1	1.57+/-0.17	100		
*hfq *P1-t1	2.42+/-0.12	66.7		
*hfq *P2-t1	5.43+/-0.07	16.9		

(*) Half-lives of transcripts were measured in strains N3433 (wt) and IBPC929 (*hfq*), transformed, when indicated, with pGEM3 empty vector, its derivative pTX367 expressing the *hfq *gene, or with plasmid pΔS15AUG bearing the constitutively expressed truncated *rpsO *gene.

(**) The *rpsO *and the *rpsO*Δ transcripts originated from the chromosome and the pΔS15AUG plasmid, respectively.

### Hfq interferes with the poly(A) dependent degradation of the *rpsO *mRNA *in vivo*

Previous investigations suggested that Hfq could affect *rpsO *mRNA stability by modulating both the poly(A)-dependent and the RNase E-mediated degradation pathways. Besides its stimulating effect on the synthesis of poly(A) tails, Hfq was shown to protect poly(A) tails from exoribonucleolytic degradation *in vitro *and to affect the length and the frequency of oligo(A) tails *in vivo *[26-29]. Moreover, Hfq was found to protect the *rpsO *mRNA from RNase E cleavage *in vitro *[29]. To evaluate the role of Hfq in the poly(A)- and the RNase E-dependent degradation pathways *in vivo*, we compared the stability of the *rpsO *transcript in *hfq*^+ ^and *hfq*-deficient strains when either of these pathways was inactivated. For this purpose, we used the *pcnB *null-mutation to fully inactivate poly(A) polymerase and the *rne*3071 allele allowing inactivation of thermosensitive RNase E at the non permissive temperature.

**Figure 2:**
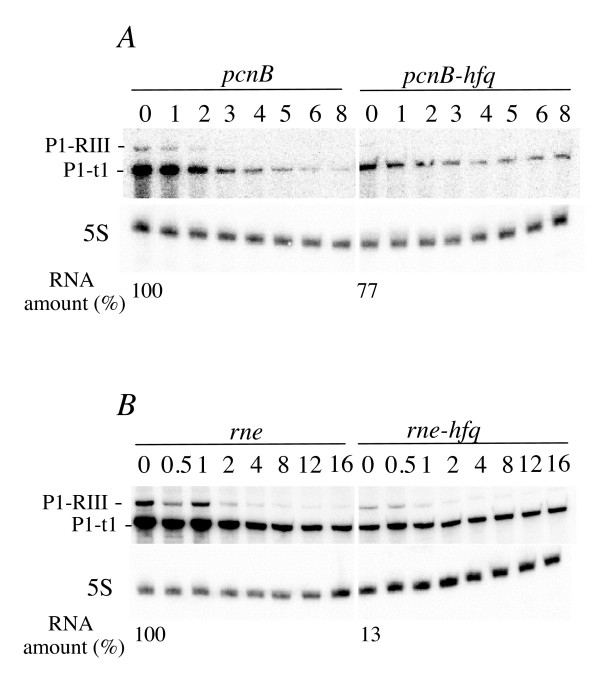
**Impact of Hfq on *rpsO *mRNA degradation pathways**. Comparison of the *rpsO *mRNA abundance and stability in *pcnB *(A) and *rne*3071 (B) mutants bearing *hfq*^+ ^or *hfq*1 alleles. (A) Cells were grown at 37°C. (B) Cells were grown at 30°C and shifted to 44°C to inactivate thermosensitive RNase E just before the addition of rifampicin. Northern-blot analysis was performed as described in the legend to Fig.1. Time intervals (min) after treating the cultures with rifampicin are indicated above the lanes. Below each Northern blots, relative amounts of the *rpsO *mRNA (normalized to 5S RNA) for 0 time (before treating with rifampicin) are indicated. Time points 0 to 4 min and 0 to 16 min. were used to calculate the half-lives in the *pcnB *mutants (Fig. 2A) and *rne *strains (Fig. 2B) respectively.

Fig. 2A shows that Hfq has no significant impact on the decay-rate of the P1-t1 *rpsO *transcript in *pcnB *cells. Half lives are 1.32 +/- 0.09 min in *hfq*^+ ^and 1.39 +/- 0.10 min in *hfq*^- ^in the first phase of the decay (time 0 to 4 min) and, in contrast to what happens in *pcnB*^+ ^cells, that *rpsO *mRNA seems to be slightly more stable in the *hfq*^- ^strain 4 min after rifampicin addition. Moreover, the drop in the *rpsO *mRNA concentration resulting from Hfq inactivation is **less marked **in the absence of PAP I (2.3 times instead of 10 times in the PAP I-containing cells).

These data suggest that Hfq does not significantly affect the RNase E dependent degradation pathway, which accounts for RNA decay in the *pcnB *null-mutant [40]. Moreover, if extended to other strains, this conclusion implies that the stabilization that occurs in *hfq*^- ^cells may not result from the impairment of the RNase E-mediated pathway. It is therefore likely, that changes of *rpsO *mRNA stability and concentration described above require active PAP I; a prediction which prompted us to verify how Hfq affects the decay-rate of the *rpsO *transcript when thermosensitive RNase E was inactivated.

We have ascertained that polyadenylation contributes to degradation of the *rpsO *mRNA in the absence of RNase E. As expected, the *rpsO *P1-t1 transcript was significantly stabilized when thermosensitive RNase E was inactivated [35]. Moreover, Hfq had a strong effect on the *rpsO *mRNA abundance. While the stability of the P1-t1 transcript rose from 6.99 +/- 0.14 min. to 11.18 +/- 0.14 min. the steady-state amount of the transcript was reduced 7.7 times in the absence of Hfq (Fig. 2B). These data reinforce the hypothesis above that Hfq deficiency has a stabilizing effect on the *rpsO *mRNA, which in theory should cause an accumulation of RNA. However, this stabilization is completely masked by a drop in mRNA concentration whose origin is independent of RNA stability. Moreover, they also confirm that the extent of the impact of Hfq on RNA stabilization and concentration depends on the presence of PAP I in the cell. It is worth noting again, that this effect is also observed in wild type strains where the degradation of the *rpsO *mRNA is mostly carried out by RNase E. It must be recalled here, that the RNase E and PAP I dependent pathways are somehow related and can substitute for one another [41].

### Hfq does not affect translation and autoregulation of the *rpsO *mRNA

Since mRNAs are generally protected by translating ribosome, we reasoned that the stabilization of the *rpsO *transcript in the Hfq deficient strains may result from an increase in translation efficiency. One could imagine that the autoregulation loop which adjusts *rpsO *translation efficiency to the need for S15, may convert the drop in mRNA resulting from Hfq deficiency into a stimulation of translation aiming to compensate for the poor levels of S15 synthesis. Alternatively, Hfq-mRNA interactions could directly affect translation or the autogeneous repression by S15.

These hypotheses led us to look at the behaviour of the constitutively translated *rpsO *transcript, which does not bear the operator recognizable by S15 and hence whose translation is independent of the S15 yield. This was achieved by using a plasmid-borne *rpsO *gene in which the first 122 nucleotides of the coding region were deleted, seven of these nucleotides being involved in the formation of the translational operator. This plasmid, referred to as pΔS15AUG, encodes a truncated S15 polypeptide unable to participate in the 30S assembly and to repress translation of the wild-type *rpsO *message [38]. The stabilities of mRNAs originating from the chromosome (*rpsO*) and from the plasmid (*rpsO*Δ) were determined in *hfq*^+ ^and *hfq*1 backgrounds. As was shown previously in wild-type cells the constitutively translated *rpsO*Δ transcript was more stable than the native *rpsO *mRNA due to higher translation levels [38]. The greater stability of the *rpsO*Δ transcript was also observed in the *hfq *mutant (Table 1). Moreover, just as in the case of the wild-type *rpsO *mRNA transcript originating from the chromosome, Hfq inactivation caused both a drop in the *rpsO*Δ mRNA level and an increase in its stability (Table 1). These data indicate that the consequences of Hfqdeficiency on mRNA level and stability do not rely on the autoregulation of S15 synthesis. Indeed, the observed stabilization of the unregulated *rpsO*Δ mRNA in the *hfq *mutant rules out the possibility that a drop in a functional *rpsO *mRNA concentration provokes increased translation efficiency through the autoregulation loop, resulting in stabilization of mRNA.

**Figure 3:**
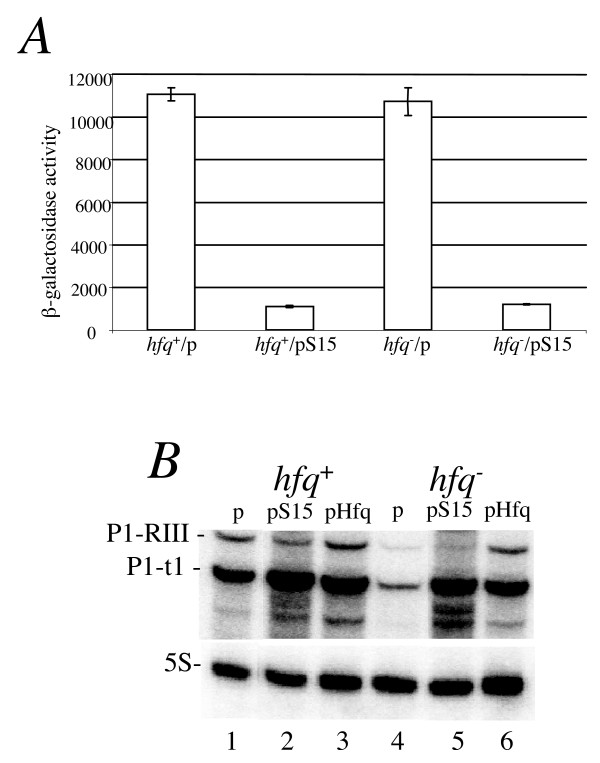
**Hfq is not implicated in autogeneous repression of the *rpsO *mRNA by S15**. (A) β-Galactosidase activities of the *rpsO'-'lacZ *chromosomal fusion in the *hfq*^+ ^and *hfq*Δ derivatives of strain IBrpsO188::*lacZ *(Materials and methods) carrying plasmids pACYC184 (p) and its derivative pS15 expressing the *rpsO *gene. (B) Northern-blot analysis of the *rpsO *mRNA in strain IBrpsO188::*lacZ *transformed with pACYC184 (p) and its derivatives pS15 and pHfq (pTX381). Lower panel shows the amount of 5S RNA per lane. RNA was extracted from cells grown at 37°C to OD_600_≈0.4 in LB supplemented with chloramphenicol (34 μg/μl).

Whether Hfq affects the autoregulation of the genuine *rpsO *mRNA bearing an intact *rpsO *operator recognizable by S15 was directly examined by using a specially generated strain in which the *rpsO *promoter and the whole translation initiation region (TIR) governed synthesis of the chimeric ß-galactosidase from the chromosomal *lacZ *gene. The *hfq*^+ ^and *hfq*^- ^isogenic variants of this strain were obtained by P1 transduction, and then each of them was transformed by a plasmid, pS15, expressing the *rpsO *gene from its own promoter and thereby serving as a source of additional S15 in a cell, or by a parent vector pACYC184 as a control. The β-galactosidase assay showed that neither the expression of the translationally active gene fusion (cells transformed by pACYC184) nor that of the translationally repressed fusion (cells transformed by pS15) were modified by the *hfq *mutation (Fig. 3A). Indeed, the impact of S15 protein expression in trans on the *rpsO-lacZ *fusion was similar in both *hfq*^+ ^and *hfq *deficient strains (in both cases the repression factor was about 9-10) (Fig. 3A). Thus, Hfq does not seem to be involved in the autogeneous control of S15 synthesis.

As one can notice (Fig. 3B), the reduction of the *rpsO *mRNA level in the *hfq*^- ^strain is more pronounced in strains with empty vector (lanes 1 and 4) than for the pS15 bearing strains (lanes 2 and 5) indicating that the gene dose plays a role in this effect. As expected, the drop in the *rpsO *mRNA abundance was complemented by the plasmid pTX381 (pHfq), a pACYC 184-derivative expressing the wild-type *hfq *gene from its own promoter.

**Figure 4:**
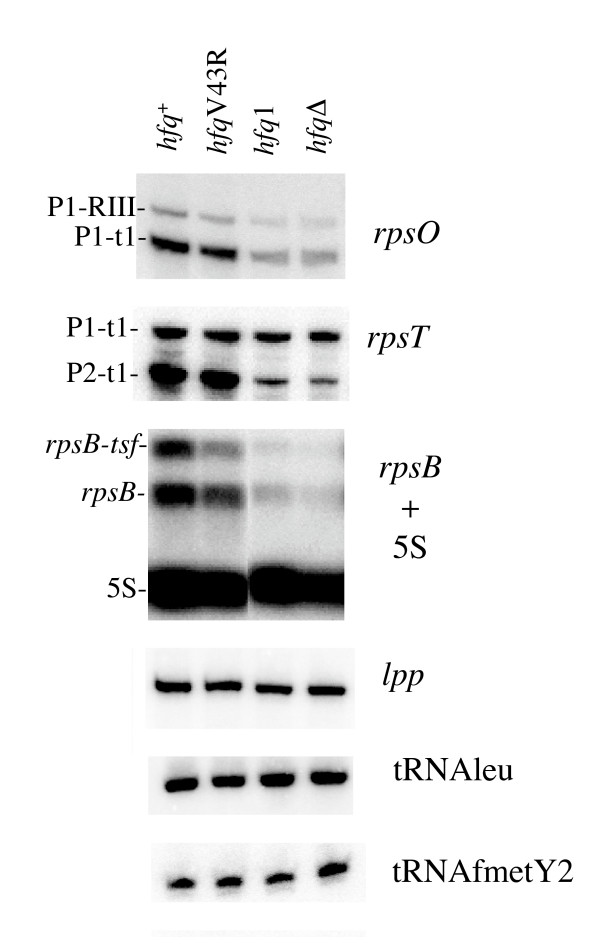
**Hfq inactivation affects levels of some but not all transcripts**. Total RNA from strains N3433 (*hfq*^+^), IBPC941 (*hfq*V43R), IBPC929 (*hfq*1), IBPC953 (*hfq*Δ) were analysed on Northern blot probed for *rpsO, rpsT, rpsB, lpp*, tRNA leu, tRNA fMetY2 and 5S rRNA

It is important to note that the changes in abundance and stability of the *rpsO *transcripts do not depend on the nature of the inactivating mutation. We found that inactivation of the *hfq *gene by insertion of a Ω-cassette (*hfq1*) or by in-frame deletion (*hfq*Δ) had similar impacts. In contrast, the V43R substitution, which only partially impairs Hfq function [16] did not significantly affect the amount of the *rpsO *mRNA in the cell, indicating that complete Hfq deficiency is required for the reduction (Fig. 4).

### Hfq selectively affects abundance of several transcripts

In order to determine whether the effect of Hfq deficiency on the *rpsO *mRNA could be also observed for other transcripts, we probed total RNA isolated from cells containing wt and mutant *hfq *alleles for *rpsT, rpsB *and *lpp *mRNAs and for the Leu1 and MetY2 tRNAs. While it has been reported that Hfq binds the *rpsO *mRNA [16,29] and tRNAs [18,19]*in vitro*, the other three transcripts were not examined. However, the fact that mRNAs from many ribosomal protein operons co-immunoprecipitate with Hfq [20] argues in favor of such a possibility for *rpsB *and *rpsT*. The Northern blots revealed that *hfq *inactivation did not modify *lpp *mRNA or tRNA abundance (Fig. 4). In contrast, the two *rpsT *mRNA species were less abundant in the *hfq *mutant, the stronger effect being observed for the P2-t1 *rpsT *transcripts initiated at promoter P2. Just as in the case of *rpsO*, the decreased level of *rpsT *transcripts was accompanied by a significant increase in their stability (Table 1).

**Figure 5:**
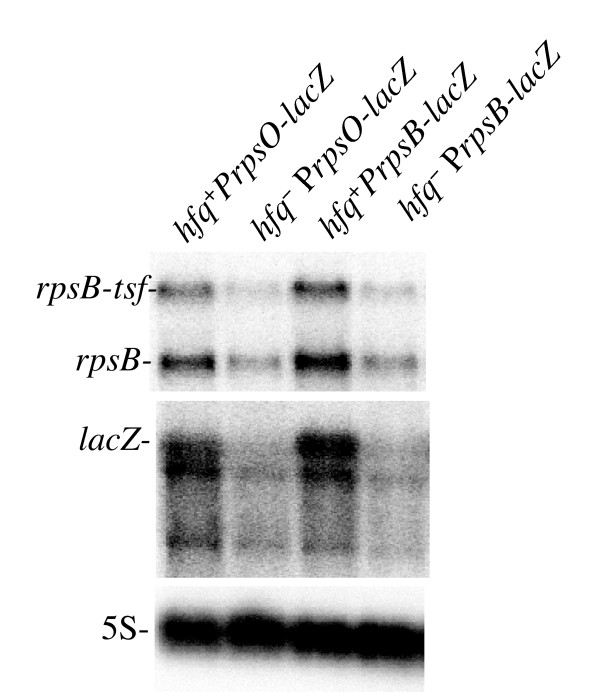
**Negative impacts of Hfq deficiency on abundance of the *rpsO-lacZ *and *rpsB-lacZ***. Preparations of total RNA from strains IBrpsO188::*lacZ *(*hfq*^+^*PrpsO-lacZ*), IBrpsO188::*lacZ hfq*Δ, (*hfq*^- ^P*rpsO-lacZ*), LABrpsB208::*lacZ *ΔGGGU (*hfq*^+^*PrpsB-lacZ*) and LABrpsB208::*lacZ *ΔGGGU *hfq*Δ (*hfq*^- ^P*rpsB-lacZ*) were analyzed on Northern blot and probed for *rpsB, lacZ *and 5S.

Transcripts of the *rpsB-tsf *operon also behave like the *rpsO *transcripts in response to Hfq deficiency. The *rpsB *and *rpsB-tsf *mRNAs are transcribed from a single *rpsB *promoter [43]. The monocistronic *rpsB *transcripts terminate at the attenuator structure in front of *tsf*, while about one third of the total transcripts read through the attenuator, generating the bicistronic *rpsB-tsf *mRNA. The levels of both transcripts were significantly decreased by Hfq deficiency (Fig. 4, 5). Moreover, like in the case of the *rpsO-lacZ *transcript, the abundance of the chimeric *rpsB-lacZ *mRNA (the transcription product from a *rpsB-lacZ *translational fusion whose expression was driven by the *rpsB *promoter and the *rpsB *TIR) was also lower in the absence of Hfq in a cell (Fig. 5). Because the *rpsB-lacZ *construction used in these experiments bears a small deletion in the *rpsB *TIR, which abolishes autogeneous regulation by S2 in trans [43], this effect is not related to changes in translation control.

**Figure 6:**
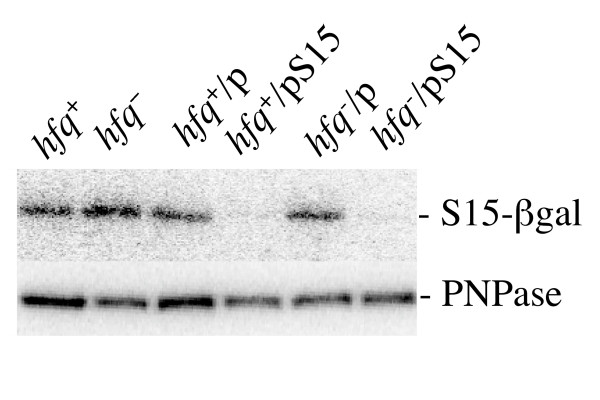
**Balanced production of proteins encoded by the *rpsO-lacZ *and *pnp *genes in *hfq***^**+**^**and *hfq***^**-**^**backgrounds**. PNPase and β-Galactosidase levels were determined by Western-blotting in the *hfq*^+ ^and *hfq*Δ derivatives of strain IBrpsO188::*lacZ*, bearing the *rpsO'-'lacZ *chromosomal fusion (Materials and methods) and carrying plasmids pACYC184 (p) or its derivative pS15 expressing the *rpsO *gene.

Interestingly, the *rpsB-lacZ *fusions produced similar level of β-galactosidase activity in *hfq*^+ ^and *hfq*Δ isogenic strains (12150 +/- 400 and 11800 +/- 250 units of β-galactosidase in the *hfq*^+ ^and *hfq*Δ strains respectively). The same was observed for the *rpsO-lacZ *fusion (Fig. 3A), where the ß-galactosidase activities in *hfq*^+ ^and *hfq*^- ^strains were similar. This means that despite the reduced levels of both mRNAs (*rpsB-lacZ *and *rpsO-lacZ*) in *hfq*^- ^mutant, the relative levels of the corresponding proteins remain about the same, indicating that the reduction in mRNA concentration may be compensated by an increase of translation efficiency or by other compensatory mechanisms. The reduced levels of many transcripts for important cellular proteins (ribosomal proteins, translation factors and others) were observed in a genome-wide transcriptome analysis of a *hfq *mutant by Guisbert et al. [12]. The drop in levels of the transcripts encoding ribosomal proteins may slow down ribosome synthesis, thereby being responsible (at least partly) for the known growth defects of the *hfq *mutants. In turn, the slower rate of ribosome synthesis should slow down the synthesis of other cellular proteins, so that finally the overall proteome of essential proteins is kept in balance. Thus, as revealed by Western-blotting (Fig. 6), the relative amounts of the ß-galactosidase produced from the *rpsO-lacZ *fusion gene and polynucleotide phosphorylase (encoding by the *pnp *transcripts whose level, as will be shown below, is independent of the presence of Hfq) remain about the same in *hfq*^+ ^and *hfq*^- ^strains despite a large reduction in the *rpsO-lacZ *transcript level caused by Hfq deficiency.

### Transcription of the *pnp *gene from its own promoter is not sensitive to *hfq *deletion

**Figure 7:**
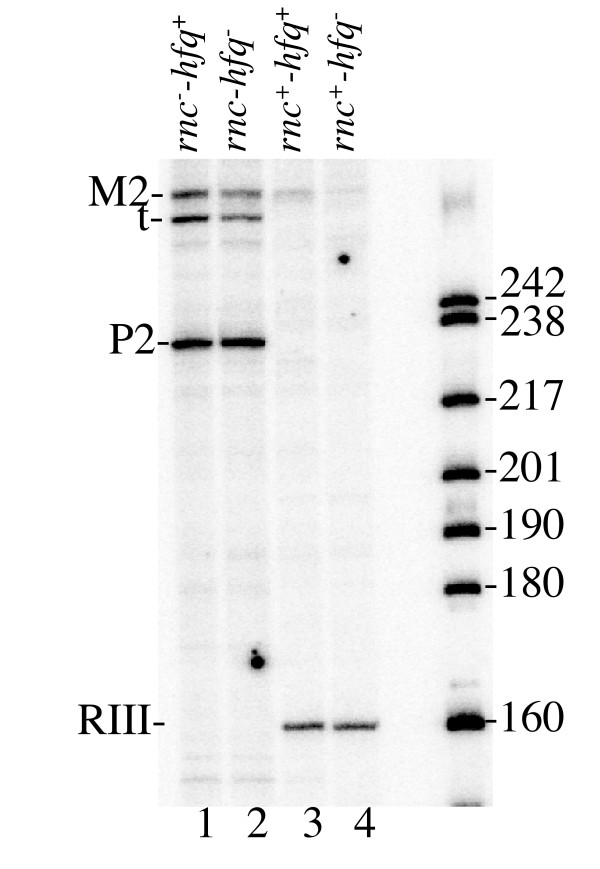
**Transcription of the *pnp *gene from the *pnp *promoter is not affected by Hfq**. Total RNA was extracted from strains N3433 (*hfq*^+^), IBPC929 (*hfq*1), IBPC633 (*rnc*^-^) and IBPC927 (*rnc*^- ^*hfq*^-^) and the 5'-extremities of the *pnp *transcripts (in equal amounts of RNA) were mapped by primer extension using a 5'-labeled oligonucleotide complementary to the *pnp *mRNA region 160 nucleotides downstream of the RNase III processing site. The right lane shows the 5'-labeled RNA size markers separated on the same gel. M2 and t bands corresponding to arrest of reverse transcription at an RNase E processing site and a stable hairpin within the *rpsO-pnp *bicistronic transcript from the *rpsO *promoter, P2 and RIII- transcripts from the *pnp *promoter unprocessed and processed by RNase III, respectively.

Since the above data suggest that changes in mRNA steady-state levels cannot be caused by modifications of mRNA stability or translation efficiency, we suspected that the lack of Hfq might exert a direct negative impact on transcription yield. Previous data have shown that Hfq may act as a DNA binding protein [30] that is able to change superhelicity of plasmid DNA [5]. Thus the changes in transcription yield observed in the absence of Hfq could be the consequence of modified DNA topology. If this is the case, the loss of Hfq should change the levels of the *pnp *mRNA, whose transcription from its own P2 promoter was reported to depend on DNA supercoiling [44]. The relative abundance of the *pnp *transcripts was deduced from the amounts of cDNA generated by primer extension. Since the *pnp *mRNA is strongly destabilized by a RNase III cleavage in the 5' UTR [34], these experiments were also performed in a RNase III deficient strain (*rnc *mutant) where RNA concentration is expected to reflect the transcription efficiency of *pnp *more directly. Fig. 7 shows that Hfq deficiency has no detectable effect on the intracellular concentrations of either the primary transcript initiated at P2 or the transcript processed by RNase III. This contrasts with the bands referred to as M2 and t at the top of the autoradiograph (Fig. 7) which correspond to arrest of reverse transcription at an RNase E processing site and a stable hairpin in the *rpsO-pnp *bicistronic transcript initiated at the *rpsO *promoter (P1). Both bands diminished in intensity in *hfq *strain, confirming the decrease in abundance of transcripts from the *rpsO *promoter observed by Northern-blot technique. Thus, the lack of Hfq affects transcription yield only from a subset of promoters (e.g. *rpsO, rpsB, rpsT*), and this negative effect is unlikely to result from changes in DNA topology. Finally, PNPase was used as a control to verify that Hfq does not globally affect protein synthesis (see above).

## Discussion

The experiments described above show that Hfq deficiency may cause modifications of both mRNA abundance and stability, but these effects are not related. Indeed, stabilization of the *rpsO *mRNA takes place even in the absence of translational regulation, which presumably occurs in response to a drop in mRNA levels in the cell, and the decrease in mRNA concentration is accompanied by RNA stabilization expected to cause its accumulation. The requirement of PAP I activity for both stabilization and decrease of *rpsO *transcripts upon Hfq inactivation suggests that polyadenylation and Hfq act synergistically. The fact that mRNA stabilization in the absence of Hfq is only observed if PAP I is active might be explained by assuming that Hfq can no longer stimulate the elongation of oligo(A) extensions used as recognition sites by exoribonucleases. At the same time, the competition between Hfq and RNase E for the same site on the *rpsO *mRNA, which was observed *in vitro *[29], does not seem to play a role in the control of mRNA degradation under conditions of exponential growth in rich medium (growth conditions used in our experiments): indeed, the RNase E pathway of decay is not affected by Hfq inactivation. We cannot explain right now why Hfq has a significant impact on the *rpsO *mRNA abundance if PAP I is active. Nevertheless, the data above showing that the drop in *rpsO *mRNA concentration does not result from Hfq-mediated posttranscriptional events led us to the conclusion that it may reflect a negative impact of the loss of Hfq on transcription yield.

Recent microarray analysis revealed down-regulation of many ribosomal protein (r-protein) operons caused by lack of Hfq [12]. Moreover, earlier work also reported the co-immunoprecipitation of Hfq with mRNAs of r-protein operon in *hfq*^+ ^cells, and their general loss in *hfq *mutants [20]. No plausible explanation for these effects was proposed. The implication of Hfq-dependent ncRNAs in positive regulation of house-keeping genes is very unlikely. The finding that a similar negative impact on abundance of the *rpsO *and *rpsB *transcripts (Fig. 3B and 4), on one hand, and on the corresponding reporter constructs *rpsO-lacZ *and *rpsB-lacZ *(Fig. 5), on another hand, implies that the observed reduction of the steady-state RNA level in *hfq*^- ^strains may result from changes in promoter recognition or other early steps of transcription.

It is unlikely that Hfq discriminates promoters directly. However, there remains a possibility that Hfq may affect promoter recognition indirectly. The *E. coli *RNA polymerase holoenzyme is composed of the core enzyme (consisting of α2, β, β' and ω subunits) tightly bound with one of seven σ subunits which determine sequence specific contacts with promoter DNA. While the level of the housekeeping σ^70 ^subunit is constant under all growth conditions, the intracellular concentrations of the six other σ subunits vary as a function of the growth phase, growth conditions and upon exposure to environmental stress [45]. There is considerable evidence for competition between sigma factors for core RNA polymerase *in vivo*; therefore alterations in the level of one sigma factor may influence the competitor properties of others [46,47]. At least two sigma factors, σ^S ^and σ^E^, are affected by Hfq inactivation, indicating that the relative abundance of different holoenzymes varies between *hfq*^+ ^and *hfq*^- ^strains. Hfq deficiency decreases *rpoS *(σ^S^) expression at the translational level [4,48]. At the same time the *rpoE *mRNA encoding σ^E ^is up-regulated in a *hfq *mutant [12], thus enhancing the competitiveness of σ^E ^for the core RNA polymerase. However, the transcription of *pnp *as that of the tRNA genes, all of which are insensitive to Hfq deficiency (Fig. 4 and 7), are σ^70^-dependent, arguing against the possibility that competition between sigma factors is responsible for the down-regulation of the r-protein mRNAs. Alternatively, it is possible that Hfq-mediated modulation of transcription may occur at an early elongation step. Indeed, during the past decade, transcription elongation has been appreciated as a regulated phase of transcription. Regulation of transcript elongation occurs mainly due to transcriptional pausing that can be stabilized by several mechanisms: hairpin-dependent pausing (e.g. well-characterized *his *leader pause site), backtracked pauses, and σ-stabilized RNA polymerase stalling downstream from promoters [49-53]. As concerns the potential role of Hfq in regulation of transcript elongation via hairpin-dependent pausing, most pause signals have been found in the leader regions of certain amino acid biosynthetic operons. The reported co-immunoprecipitation of Hfq with mRNAs encoding leader peptides of these operons may be indicative of a role of Hfq in modulating attenuation [20]. Moreover, this role should be positive, as microarray analysis revealed that lack of Hfq decreased the levels of mRNAs implicated in threonine *(thrABC*) or histidine (*hisG, D, C, B, H*) biosynthesis pathways regulated by attenuation [12].

The positive role for Hfq in transcription *in vivo *we propose here is consistent with a reported stimulatory effect of Hfq on the overall yield of transcription *in vitro *[23]. Hfq may play a chaperoning role in co-transcriptional folding of a nascent RNA chain, counteracting transcription pausing or arrest and preventing premature release of the transcript. Biologically important RNAs often solve their folding problem using the assistance of chaperone and cofactor proteins [54]. In this respect, it should be mentioned that folding of long structured mRNA leaders of the *rpsO *and *rpsB *operons plays a crucial role in regulation of their expression [43,55]. In addition to the folding problems, transcriptional pauses within the mRNA leaders of r-protein mRNAs may also be stabilized by rebinding of σ^70 ^to the elongating RNA polymerase, when -10-like elements located downstream from promoters are exposed in transcriptional bubble [50,52,53]. Indeed, -10-like elements followed by three GC base pairs (consensus for σ^70^-dependent pausing) are easily recognized within the *rpsO *(TACACTGGG, positions +40 to +48 from transcription start) and *rpsB *(TAATATGGG, positions +110 to +118) mRNA leaders. Taking this into account, it is tempting to suppose that Hfq may play a positive role in the co-transcriptional folding and/or maintaining of the nascent RNA chain during transcriptional pauses, preventing transcription arrest and formation of abortive elongation complex. If this is the case, the portion of abortive transcription on the pause-inducing sites should increase in the absence of Hfq in the cell, leading to a reduced steady-state level of the mRNAs produced. The mRNA transcripts that escape abortive events will be less abundant but more stable either because they are translated more efficiently to produce sufficient amount of r-proteins for ribosome biosynthesis, or due to inefficiency of poly(A)-dependent degradation pathway mediated by Hfq. We believe that the proposed here potential role of Hfq in maintaining nascent transcripts to prevent premature transcript release at the pausing sites is the most plausible explanation for the observed down-regulation of r-protein mRNAs in *hfq *null-mutants.

## Conclusions

We present here several lines of evidence indicating that *rpsO, rpsB, rpsB-tsf *and *rpsT *mRNAs are down-regulated in *hfq *null mutants and that the reduction of the mRNA levels upon Hfq deficiency cannot be explained by destabilization of mRNAs and relates rather to changes in transcription efficiency. Our results provide an explanation for the recent microarray analysis which has revealed down-regulation of several ribosomal protein (r-protein) operons caused by the lack of Hfq. Taken together, Hfq appears to have a much greater role in RNA metabolism than previously anticipated involving all the steps of an RNA molecule's "life", from its synthesis to its degradation.

## Methods

### Bacterial strains and plasmids

The name and origin of the strains used here are listed in Table 2. A DNA fragment comprising the *rpsO *regulatory regions (*rpsO *promoter and translation initiation region, positions from -188 to +58 with respect to A+1 in the initiator ATG) was amplified from the genomic *E. coli *DNA and inserted into pEMBLΔ 46/BamHI, HindIII in frame with the *lacZ *coding sequence [56]. Primers used for PCR amplification were: PrpsO-for 5'-

CGT*GGATCC*TCGTCGCCTGGTGGTTG corresponding to the sequence from -188 to -162 upstream of *rpsO *and bearing the BamHI site near the 5'-terminus (italicized) and TIRrpsO-rev 5'-CAG*AAGCTT*GCGTCACGACCAAACTC complementary to the *rpsO *coding sequence from +40 to +58 and bearing the HindIII site (italicized). The resulting plasmid pErpsO188 was then used to create the ENSO derivative IBrpsO188::*lacZ *by homologous recombination [56]. This strain was used to generate two tetracycline-resistant derivatives *hfq*^+ ^and *hfq*Δ by P1 transduction as described [16].

**Table 2:** *Escherichia coli *strains and plasmids used in this work

Strain or plasmid	Relevant characteristics	Reference/source
Strains		
N3433	HfrH*lacZ43 relA1 spoT1 thi-1*	D. Apirion
N3431	N3433 *rne3071(ts)*	D. Apirion
IBPC633	N3433 *rnc105*	[34]
IBPC903	N3433 Δ*pcnB*(kan^R^)	[58]
IBPC927	IBPC633 *hfq1*::Ω(kan^R^, *Bcl*I)	this work
IBPC928	N3431 *hfq1*::Ω(kan^R^, *Bcl*I)	this work
IBPC929	N3433 *hfq1*::Ω(kan^R^, *Bcl*I)	[16]
IBPC941	N3433 *hfq*V43R *cycA30*::Tn*10*	[16]
IBPC953	N3433 *hfq*Δ22-294 *cycA30*::Tn*10*	[16]
IBPC981	IBPC903 *hfq*Δ22-294 *cycA30*::Tn*10*	this work
ENSO	HfrG6*lacZ*Δ12	[56]
IBrpsO188::lacZ(*)	ENSO *rpsO'-'lacZ*	this work
LABrpsB208ΔGGGU(*)	ENSO *rpsB'-'lacZ *bearing deletion in	
	*rpsB *TIR from -72 to -69	[43]
Plasmids		
pΔS15AUG	pCL1921 derivative expressing Δ*rpsO*	[38]
pACYC184	Tet^r^, Cm^r^	[59]
pS15	pACYC184 derivative expressing *rpsO*	this work
pGEM3	Amp^r^	Promega corp.
pTX367 (**)	pGEM3 derivative expressing *hfq*	[5]
pTX381 (**)	pACYC184 derivative expressing *hfq*	[5]

(*) Tetracyclin-resistant derivatives of these strains, bearing *cycA30*::Tn*10 *and *hfq*Δ22-294 *cycA30*::Tn*10*, were constructed for using in this work. (**) These two plasmids contain the same insert. They were used without distinction assuming that the same amount of Hfq is overproduced due to autoregulation [16,17].

The plasmid pS15 was created by cloning the whole *rpsO *gene flanked with its own promoter and terminator into BamHI and HindIII sites of pACYC184. The DNA fragment to be cloned was generated by PCR on *E. coli *genomic DNA using rpsO-for corresponding to the positions (-149) to (-126) relative to the *rpsO *translation start and bearing the BamHI site; 5'-CAG*GGATCC*GTCTTGCGATAACAG and rpsO-rev, complementary to the positions from +303 to +325 of the *rpsO *mRNA and bearing the HindIII site 5'-CCGT*AAGCTT*GAAAAAAGGGGCC.

Construction of strains LABrpsB208::*lacZ *carrying chromosomal *rpsB'-'lacZ *fusion with the *rpsB *portion comprising the *rpsB *promoter and TIR (positions from -208 to +41 relatively the initiator ATG codon) was described [43]. Here, we used a derivative of this strain, LAB*rpsB208*::*lacZ *ΔGGGU bearing a small deletion in the *rpsB *5'-untranslated region (from -72 to -69), which abolishes autoregulation [43]. The *hfq*Δ allele [16] was transferred into this derivative by P1 transduction to generate *hfq*^+ ^anf Δ*hfq *variants.

### Cell growth and β-galactosidase assay

In the case of IBrpsO188::*lacZ *and LABrpsB208::*lacZ*, cell cultures were grown at 37°C in LB medium supplemented if necessary with tetracycline (12 μg/ml) or chloramphenicol (35 μg/ml), in the absence of IPTG (*lac*-promoter is closed, *rpsO *or *rpsB *promoters are active). Cells were harvested in exponential phase (OD_600_≈0.4-0.5). The β-galactosidase activity was measured in clarified cell lysates [54] and expressed in nmol ONPG (*o*-nitrophenyl-b-D-galactopiranoside) hydrolysed per minute per milligram of total soluble proteins. Protein concentration in lysates was measured by Bradford assay (Bio-Rad).

### Western blotting

Cellular proteins (20 μg) were separated on a 12% SDS-PAGE gel and analyzed with the use of ^125^I-iodinated A-protein as described in [16]. Polyclonal rabbit antibodies raised against β-galactosidase (ICN Biomedicals, Inc.) and PNPase (kindly provided by A. J. Carpousis) were used.

### RNA extraction and analysis

RNAs were prepared form bacteria grown to an A_650 _= 0.35-0.4, according to [37]. Templates for the synthesis of *rpsO *and *rpsT *RNA probes were obtained by PCR amplification as described [57]. The *lacZ *template was obtained using *TAATACGACTCACTATAGGG*ATACTGACGAAAC and GCCGTCGTTTTACAACGTC, the *lpp *probe using *TAATACGACTCACTATAGGG*TATTTAGTAGCCATGTTG and GTTCTACTCTGCTGGCAG, and the *rpsB *probe using *TAATACGACTCACTATAGGG*TTCACGAAGAACTGGTCG and ATGGCAACTGTTTCCATGCG oligonucleotides as primers. The upstream primer includes the T7 promoter indicated in italics. RNA probes were synthesized by T7 RNA polymerase yielding uniformly labeled RNAs with [α-^32^P] UTP [26]. 5S rRNA, tRNALeu1 and tRNAMetY were probed with the 5'-labelled oligonucleotides 5'-ACTACCATCGGCGCTACGGC, 5'-CCCCCACGTCCGTAAGGACA and 5'-CGGGTTATGAGCCCGACGA, correspondingly.

Total RNA separation on 1% agarose formaldehyde gel or acrylamide/urea gel and Northern blotting were previously described [34,35]. RNA levels were quantified using a PhosphoImager, and values were corrected for variations in RNA loading by hybridizing the same blot for the 5S rRNA.

## Authors' contributions

JLD, IVB and EH performed all presented experiments, IVB and EH conceived the study and analyzed the data. IVB, PR and EH wrote the manuscript. All authors read and approved the final manuscript.
